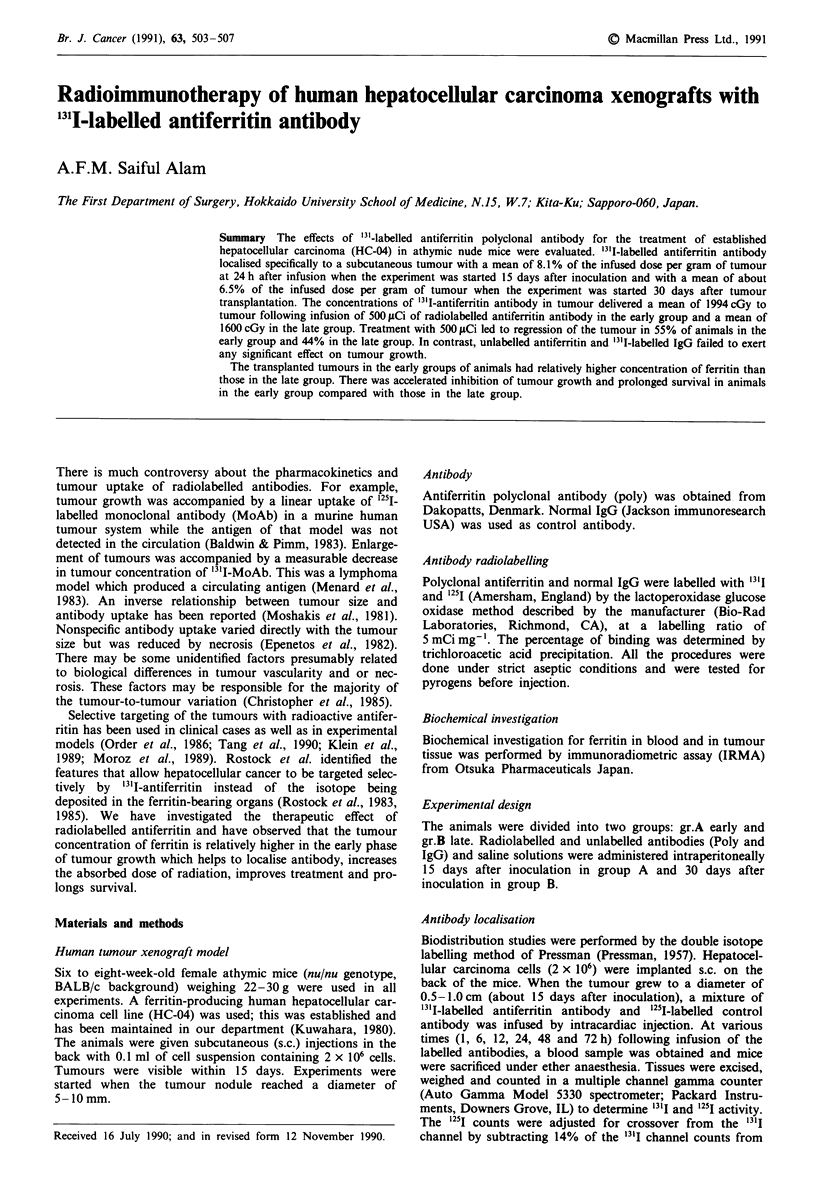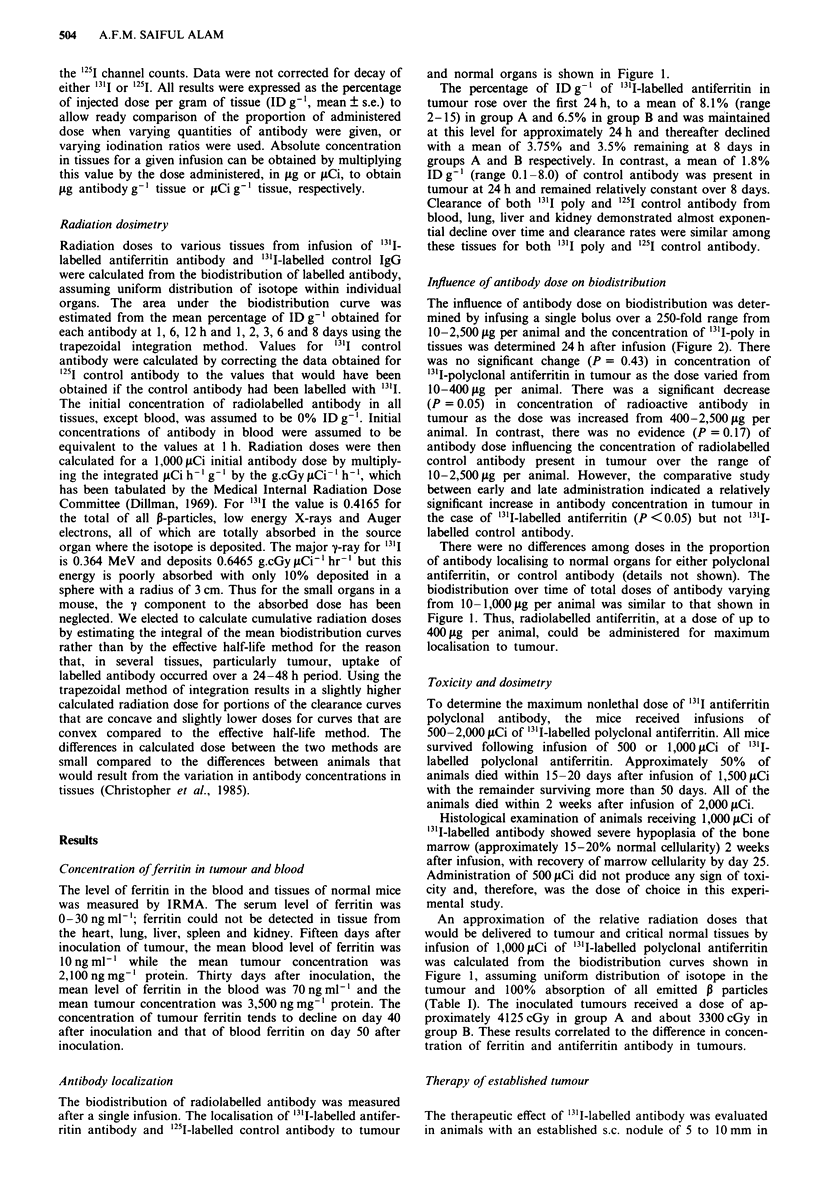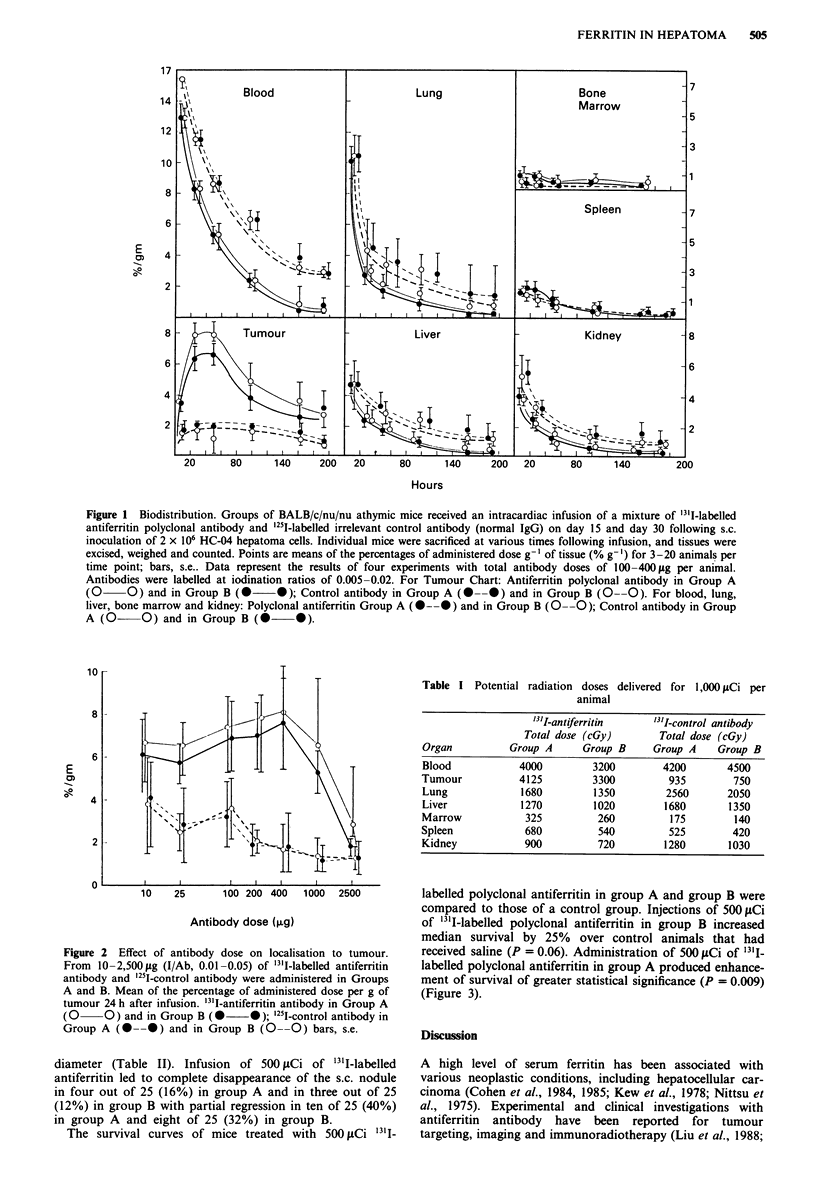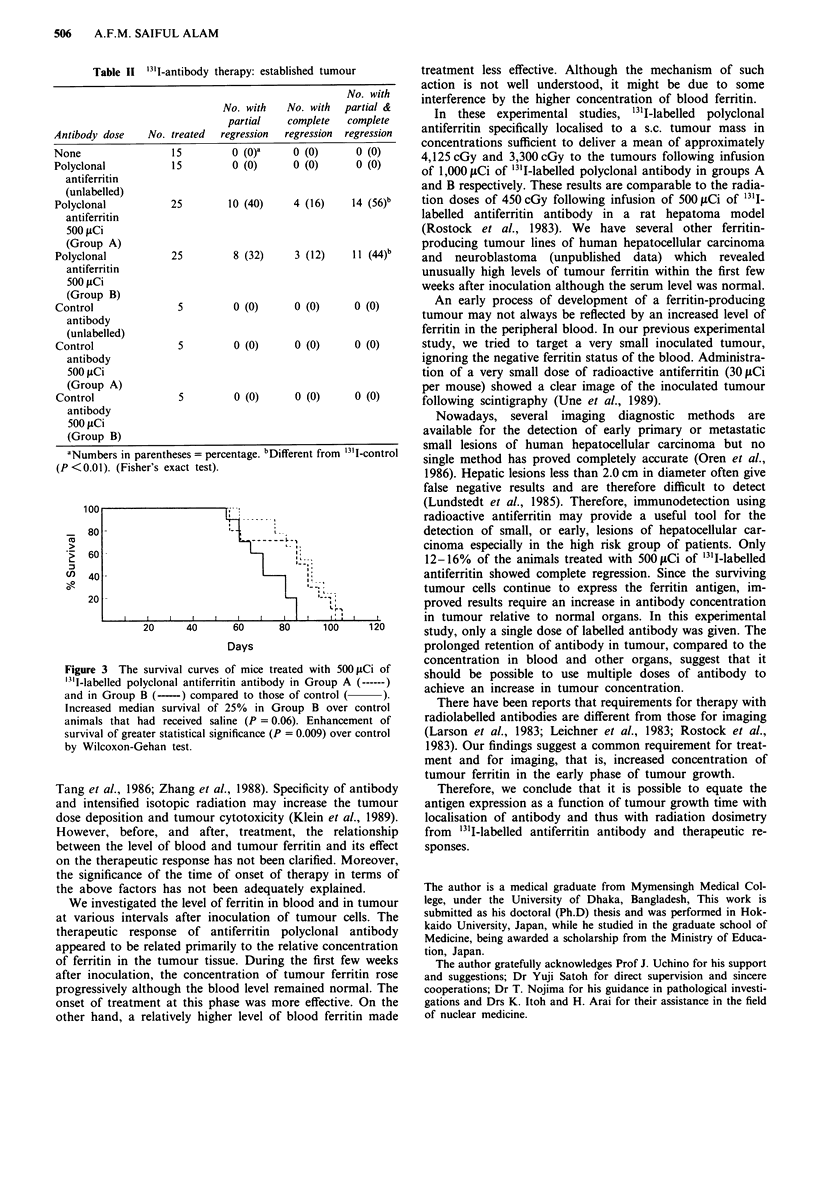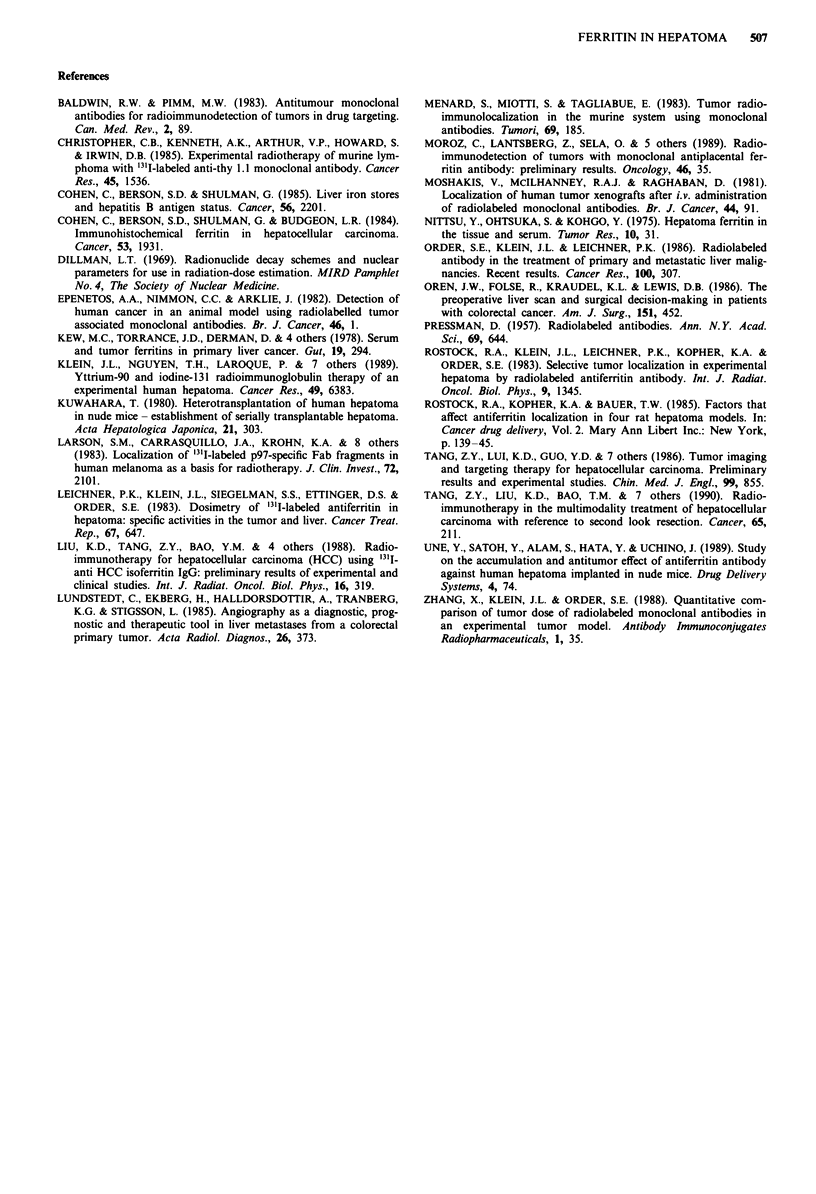# Radioimmunotherapy of human hepatocellular carcinoma xenografts with 131I-labelled antiferritin antibody.

**DOI:** 10.1038/bjc.1991.120

**Published:** 1991-04

**Authors:** A. F. Saiful Alam

**Affiliations:** First Department of Surgery, Hokkaido University School of Medicine, Sapporo, Japan.

## Abstract

The effects of 131-labelled antiferritin polyclonal antibody for the treatment of established hepatocellular carcinoma (HC-04) in athymic nude mice were evaluated. 131I-labelled antiferritin antibody localised specifically to a subcutaneous tumour with a mean of 8.1% of the infused dose per gram of tumour at 24 h after infusion when the experiment was started 15 days after inoculation and with a mean of about 6.5% of the infused dose per gram of tumour when the experiment was started 30 days after tumour transplantation. The concentrations of 131I-antiferritin antibody in tumour delivered a mean of 1994 cGy to tumour following infusion of 500 microCi of radiolabelled antiferritin antibody in the early group and a mean of 1600 cGy in the late group. Treatment with 500 microCi led to regression of the tumour in 55% of animals in the early group and 44% in the late group. In contrast, unlabelled antiferritin and 131I-labelled IgG failed to exert any significant effect on tumour growth. The transplanted tumours in the early groups of animals had relatively higher concentration of ferritin than those in the late group. There was accelerated inhibition of tumour growth and prolonged survival in animals in the early group compared with those in the late group.


Br. J. Cancer (1991), 63, 503-507 © Macmillan Press Ltd., 1991

### Radioimmunotherapy of human hepatocellular carcinoma xenografts with $^{131}$I-labelled antiferritin antibody

A.F.M. Saiful Alam

*The First Department of Surgery, Hokkaido University School of Medicine, N.15, W.7; Kita-Ku; Sapporo-060, Japan.*

**Summary** The effects of $^{131}$-labelled antiferritin polyclonal antibody for the treatment of established hepatocellular carcinoma (HC-04) in athymic nude mice were evaluated. $^{131}$I-labelled antiferritin antibody localised specifically to a subcutaneous tumour with a mean of 8.1% of the infused dose per gram of tumour at 24 h after infusion when the experiment was started 15 days after inoculation and with a mean of about 6.5% of the infused dose per gram of tumour when the experiment was started 30 days after tumour transplantation. The concentrations of $^{131}$I-antiferritin antibody in tumour delivered a mean of 1994 cGy to tumour following infusion of 500 µCi of radiolabelled antiferritin antibody in the early group and a mean of 1600 cGy in the late group. Treatment with 500 µCi led to regression of the tumour in 55% of animals in the early group and 44% in the late group. In contrast, unlabelled antiferritin and $^{131}$I-labelled IgG failed to exert any significant effect on tumour growth.

The transplanted tumours in the early groups of animals had relatively higher concentration of ferritin than those in the late group. There was accelerated inhibition of tumour growth and prolonged survival in animals in the early group compared with those in the late group.

There is much controversy about the pharmacokinetics and tumour uptake of radiolabelled antibodies. For example, tumour growth was accompanied by a linear uptake of $^{125}$I-labelled monoclonal antibody (MoAb) in a murine human tumour system while the antigen of that model was not detected in the circulation (Baldwin & Pimm, 1983). Enlargement of tumours was accompanied by a measurable decrease in tumour concentration of $^{131}$I-MoAb. This was a lymphoma model which produced a circulating antigen (Menard *et al.*, 1983). An inverse relationship between tumour size and antibody uptake has been reported (Moshakis *et al.*, 1981). Nonspecific antibody uptake varied directly with the tumour size but was reduced by necrosis (Epenetos *et al.*, 1982). There may be some unidentified factors presumably related to biological differences in tumour vascularity and or necrosis. These factors may be responsible for the majority of the tumour-to-tumour variation (Christopher *et al.*, 1985).

Selective targeting of the tumours with radioactive antiferritin has been used in clinical cases as well as in experimental models (Order *et al.*, 1986; Tang *et al.*, 1990; Klein *et al.*, 1989; Moroz *et al.*, 1989). Rostock *et al.* identified the features that allow hepatocellular cancer to be targeted selectively by $^{131}$I-antiferritin instead of the isotope being deposited in the ferritin-bearing organs (Rostock *et al.*, 1983, 1985). We have investigated the therapeutic effect of radiolabelled antiferritin and have observed that the tumour concentration of ferritin is relatively higher in the early phase of tumour growth which helps to localise antibody, increases the absorbed dose of radiation, improves treatment and prolongs survival.

### Materials and methods

### *Human tumour xenograft model*

Six to eight-week-old female athymic mice (*nu/nu* genotype, BALB/c background) weighing 22-30 g were used in all experiments. A ferritin-producing human hepatocellular carcinoma cell line (HC-04) was used; this was established and has been maintained in our department (Kuwahara, 1980). The animals were given subcutaneous (s.c.) injections in the back with 0.1 ml of cell suspension containing $2 \times 10^6$ cells. Tumours were visible within 15 days. Experiments were started when the tumour nodule reached a diameter of 5-10 mm.

Received 16 July 1990; and in revised form 12 November 1990.

### *Antibody*

Antiferritin polyclonal antibody (poly) was obtained from Dakopatts, Denmark. Normal IgG (Jackson immunoresearch USA) was used as control antibody.

### *Antibody radiolabelling*

Polyclonal antiferritin and normal IgG were labelled with $^{131}$I and $^{125}$I (Amersham, England) by the lactoperoxidase glucose oxidase method described by the manufacturer (Bio-Rad Laboratories, Richmond, CA), at a labelling ratio of 5 mCi mg$^{-1}$. The percentage of binding was determined by trichloroacetic acid precipitation. All the procedures were done under strict aseptic conditions and were tested for pyrogens before injection.

### *Biochemical investigation*

Biochemical investigation for ferritin in blood and in tumour tissue was performed by immunoradiometric assay (IRMA) from Otsuka Pharmaceuticals Japan.

### *Experimental design*

The animals were divided into two groups: gr.A early and gr.**B** late. Radiolabelled and unlabelled antibodies (Poly and IgG) and saline solutions were administered intraperitoneally 15 days after inoculation in group A and 30 days after inoculation in group B.

### *Antibody localisation*

Biodistribution studies were performed by the double isotope labelling method of Pressman (Pressman, 1957). Hepatocellular carcinoma cells ($2 \times 10^6$) were implanted s.c. on the back of the mice. When the tumour grew to a diameter of 0.5-1.0 cm (about 15 days after inoculation), a mixture of $^{131}$I-labelled antiferritin antibody and $^{125}$I-labelled control antibody was infused by intracardiac injection. At various times (1, 6, 12, 24, 48 and 72 h) following infusion of the labelled antibodies, a blood sample was obtained and mice were sacrificed under ether anaesthesia. Tissues were excised, weighed and counted in a multiple channel gamma counter (Auto Gamma Model 5330 spectrometer; Packard Instruments, Downers Grove, IL) to determine $^{131}$I and $^{125}$I activity. The $^{125}$I counts were adjusted for crossover from the $^{131}$I channel by subtracting 14% of the $^{131}$I channel counts from the $^{125}I$ channel counts. Data were not corrected for decay of either $^{131}I$ or $^{125}I$. All results were expressed as the percentage of injected dose per gram of tissue (ID $g^{-1}$, mean ± s.e.) to allow ready comparison of the proportion of administered dose when varying quantities of antibody were given, or varying iodination ratios were used. Absolute concentration in tissues for a given infusion can be obtained by multiplying this value by the dose administered, in μg or μCi, to obtain μg antibody $g^{-1}$ tissue or μCi $g^{-1}$ tissue, respectively.

*Radiation dosimetry*

Radiation doses to various tissues from infusion of $^{131}I$-labelled antiferritin antibody and $^{131}I$-labelled control IgG were calculated from the biodistribution of labelled antibody, assuming uniform distribution of isotope within individual organs. The area under the biodistribution curve was estimated from the mean percentage of ID $g^{-1}$ obtained for each antibody at 1, 6, 12 h and 1, 2, 3, 6 and 8 days using the trapezoidal integration method. Values for $^{131}I$ control antibody were calculated by correcting the data obtained for $^{125}I$ control antibody to the values that would have been obtained if the control antibody had been labelled with $^{131}I$. The initial concentration of radiolabelled antibody in all tissues, except blood, was assumed to be 0% ID $g^{-1}$. Initial concentrations of antibody in blood were assumed to be equivalent to the values at 1 h. Radiation doses were then calculated for a 1,000 μCi initial antibody dose by multiplying the integrated μCi $h^{-1}$ $g^{-1}$ by the g.cGy μ$Ci^{-1}$ $h^{-1}$, which has been tabulated by the Medical Internal Radiation Dose Committee (Dillman, 1969). For $^{131}I$ the value is 0.4165 for the total of all β-particles, low energy X-rays and Auger electrons, all of which are totally absorbed in the source organ where the isotope is deposited. The major γ-ray for $^{131}I$ is 0.364 MeV and deposits 0.6465 g.cGy μ$Ci^{-1}$ $hr^{-1}$ but this energy is poorly absorbed with only 10% deposited in a sphere with a radius of 3 cm. Thus for the small organs in a mouse, the γ component to the absorbed dose has been neglected. We elected to calculate cumulative radiation doses by estimating the integral of the mean biodistribution curves rather than by the effective half-life method for the reason that, in several tissues, particularly tumour, uptake of labelled antibody occurred over a 24–48 h period. Using the trapezoidal method of integration results in a slightly higher calculated radiation dose for portions of the clearance curves that are concave and slightly lower doses for curves that are convex compared to the effective half-life method. The differences in calculated dose between the two methods are small compared to the differences between animals that would result from the variation in antibody concentrations in tissues (Christopher *et al.*, 1985).

### Results

*Concentration of ferritin in tumour and blood*

The level of ferritin in the blood and tissues of normal mice was measured by IRMA. The serum level of ferritin was 0–30 ng $ml^{-1}$; ferritin could not be detected in tissue from the heart, lung, liver, spleen and kidney. Fifteen days after inoculation of tumour, the mean blood level of ferritin was 10 ng $ml^{-1}$ while the mean tumour concentration was 2,100 ng $mg^{-1}$ protein. Thirty days after inoculation, the mean level of ferritin in the blood was 70 ng $ml^{-1}$ and the mean tumour concentration was 3,500 ng $mg^{-1}$ protein. The concentration of tumour ferritin tends to decline on day 40 after inoculation and that of blood ferritin on day 50 after inoculation.

*Antibody localization*

The biodistribution of radiolabelled antibody was measured after a single infusion. The localisation of $^{131}I$-labelled antiferritin antibody and $^{125}I$-labelled control antibody to tumour and normal organs is shown in Figure 1.

The percentage of ID $g^{-1}$ of $^{131}I$-labelled antiferritin in tumour rose over the first 24 h, to a mean of 8.1% (range 2–15) in group A and 6.5% in group B and was maintained at this level for approximately 24 h and thereafter declined with a mean of 3.75% and 3.5% remaining at 8 days in groups A and B respectively. In contrast, a mean of 1.8% ID $g^{-1}$ (range 0.1–8.0) of control antibody was present in tumour at 24 h and remained relatively constant over 8 days. Clearance of both $^{131}I$ poly and $^{125}I$ control antibody from blood, lung, liver and kidney demonstrated almost exponential decline over time and clearance rates were similar among these tissues for both $^{131}I$ poly and $^{125}I$ control antibody.

*Influence of antibody dose on biodistribution*

The influence of antibody dose on biodistribution was determined by infusing a single bolus over a 250-fold range from 10–2,500 μg per animal and the concentration of $^{131}I$-poly in tissues was determined 24 h after infusion (Figure 2). There was no significant change ($P = 0.43$) in concentration of $^{131}I$-polyclonal antiferritin in tumour as the dose varied from 10–400 μg per animal. There was a significant decrease ($P = 0.05$) in concentration of radioactive antibody in tumour as the dose was increased from 400–2,500 μg per animal. In contrast, there was no evidence ($P = 0.17$) of antibody dose influencing the concentration of radiolabelled control antibody present in tumour over the range of 10–2,500 μg per animal. However, the comparative study between early and late administration indicated a relatively significant increase in antibody concentration in tumour in the case of $^{131}I$-labelled antiferritin ($P < 0.05$) but not $^{131}I$-labelled control antibody.

There were no differences among doses in the proportion of antibody localising to normal organs for either polyclonal antiferritin, or control antibody (details not shown). The biodistribution over time of total doses of antibody varying from 10–1,000 μg per animal was similar to that shown in Figure 1. Thus, radiolabelled antiferritin, at a dose of up to 400 μg per animal, could be administered for maximum localisation to tumour.

*Toxicity and dosimetry*

To determine the maximum nonlethal dose of $^{131}I$ antiferritin polyclonal antibody, the mice received infusions of 500–2,000 μCi of $^{131}I$-labelled polyclonal antiferritin. All mice survived following infusion of 500 or 1,000 μCi of $^{131}I$-labelled polyclonal antiferritin. Approximately 50% of animals died within 15–20 days after infusion of 1,500 μCi with the remainder surviving more than 50 days. All of the animals died within 2 weeks after infusion of 2,000 μCi.

Histological examination of animals receiving 1,000 μCi of $^{131}I$-labelled antibody showed severe hypoplasia of the bone marrow (approximately 15–20% normal cellularity) 2 weeks after infusion, with recovery of marrow cellularity by day 25. Administration of 500 μCi did not produce any sign of toxicity and, therefore, was the dose of choice in this experimental study.

An approximation of the relative radiation doses that would be delivered to tumour and critical normal tissues by infusion of 1,000 μCi of $^{131}I$-labelled polyclonal antiferritin was calculated from the biodistribution curves shown in Figure 1, assuming uniform distribution of isotope in the tumour and 100% absorption of all emitted β particles (Table I). The inoculated tumours received a dose of approximately 4125 cGy in group A and about 3300 cGy in group B. These results correlated to the difference in concentration of ferritin and antiferritin antibody in tumours.

*Therapy of established tumour*

The therapeutic effect of $^{131}I$-labelled antibody was evaluated in animals with an established s.c. nodule of 5 to 10 mm in

**Figure 1** Biodistribution. Groups of BALB/c/nu/nu athymic mice received an intracardiac infusion of a mixture of $^{131}$I-labelled antiferritin polyclonal antibody and $^{125}$I-labelled irrelevant control antibody (normal IgG) on day 15 and day 30 following s.c. inoculation of $2 \times 10^6$ HC-04 hepatoma cells. Individual mice were sacrificed at various times following infusion, and tissues were excised, weighed and counted. Points are means of the percentages of administered dose $g^{-1}$ of tissue (% $g^{-1}$) for 3–20 animals per time point; bars, s.e.. Data represent the results of four experiments with total antibody doses of 100–400 µg per animal. Antibodies were labelled at iodination ratios of 0.005–0.02. For Tumour Chart: Antiferritin polyclonal antibody in Group A (○——○) and in Group B (●——●); Control antibody in Group A (●--●) and in Group B (○--○). For blood, lung, liver, bone marrow and kidney: Polyclonal antiferritin Group A (●--●) and in Group B (○--○); Control antibody in Group A (○——○) and in Group B (●——●).

**Figure 2** Effect of antibody dose on localisation to tumour. From 10–2,500 µg (I/Ab, 0.01–0.05) of $^{131}$I-labelled antiferritin antibody and $^{125}$I-control antibody were administered in Groups A and B. Mean of the percentage of administered dose per g of tumour 24 h after infusion. $^{131}$I-antiferritin antibody in Group A (○——○) and in Group B (●——●); $^{125}$I-control antibody in Group A (●--●) and in Group B (○--○) bars, s.e.

diameter (Table II). Infusion of 500 µCi of $^{131}$I-labelled antiferritin led to complete disappearance of the s.c. nodule in four out of 25 (16%) in group A and in three out of 25 (12%) in group B with partial regression in ten of 25 (40%) in group A and eight of 25 (32%) in group B.

The survival curves of mice treated with 500 µCi $^{131}$I-labelled polyclonal antiferritin in group A and group B were compared to those of a control group. Injections of 500 µCi of $^{131}$I-labelled polyclonal antiferritin in group B increased median survival by 25% over control animals that had received saline ($P = 0.06$). Administration of 500 µCi of $^{131}$I-labelled polyclonal antiferritin in group A produced enhancement of survival of greater statistical significance ($P = 0.009$) (Figure 3).

**Table I** Potential radiation doses delivered for 1,000 µCi per animal

| | *$^{131}$I-antiferritin Total dose (cGy)* | | *$^{131}$I-control antibody Total dose (cGy)* | |
|---|---|---|---|---|
| *Organ* | *Group A* | *Group B* | *Group A* | *Group B* |
| Blood | 4000 | 3200 | 4200 | 4500 |
| Tumour | 4125 | 3300 | 935 | 750 |
| Lung | 1680 | 1350 | 2560 | 2050 |
| Liver | 1270 | 1020 | 1680 | 1350 |
| Marrow | 325 | 260 | 175 | 140 |
| Spleen | 680 | 540 | 525 | 420 |
| Kidney | 900 | 720 | 1280 | 1030 |

### Discussion

A high level of serum ferritin has been associated with various neoplastic conditions, including hepatocellular carcinoma (Cohen *et al.*, 1984, 1985; Kew *et al.*, 1978; Nittsu *et al.*, 1975). Experimental and clinical investigations with antiferritin antibody have been reported for tumour targeting, imaging and immunoradiotherapy (Liu *et al.*, 1988;

**Table II** $^{131}$I-antibody therapy: established tumour

| *Antibody dose* | *No. treated* | *No. with partial regression* | *No. with complete regression* | *No. with partial & complete regression* |
|---|---|---|---|---|
| None | 15 | 0 (0)[a] | 0 (0) | 0 (0) |
| Polyclonal antiferritin (unlabelled) | 15 | 0 (0) | 0 (0) | 0 (0) |
| Polyclonal antiferritin 500 μCi (Group A) | 25 | 10 (40) | 4 (16) | 14 (56)[b] |
| Polyclonal antiferritin 500 μCi (Group B) | 25 | 8 (32) | 3 (12) | 11 (44)[b] |
| Control antibody (unlabelled) | 5 | 0 (0) | 0 (0) | 0 (0) |
| Control antibody 500 μCi (Group A) | 5 | 0 (0) | 0 (0) | 0 (0) |
| Control antibody 500 μCi (Group B) | 5 | 0 (0) | 0 (0) | 0 (0) |

[a]Numbers in parentheses = percentage. [b]Different from $^{131}$I-control ($P < 0.01$). (Fisher's exact test).

**Figure 3** The survival curves of mice treated with 500 μCi of $^{131}$I-labelled polyclonal antiferritin antibody in Group A (------) and in Group B (——) compared to those of control (———). Increased median survival of 25% in Group B over control animals that had received saline ($P = 0.06$). Enhancement of survival of greater statistical significance ($P = 0.009$) over control by Wilcoxon-Gehan test.

Tang *et al.*, 1986; Zhang *et al.*, 1988). Specificity of antibody and intensified isotopic radiation may increase the tumour dose deposition and tumour cytotoxicity (Klein *et al.*, 1989). However, before, and after, treatment, the relationship between the level of blood and tumour ferritin and its effect on the therapeutic response has not been clarified. Moreover, the significance of the time of onset of therapy in terms of the above factors has not been adequately explained.

We investigated the level of ferritin in blood and in tumour at various intervals after inoculation of tumour cells. The therapeutic response of antiferritin polyclonal antibody appeared to be related primarily to the relative concentration of ferritin in the tumour tissue. During the first few weeks after inoculation, the concentration of tumour ferritin rose progressively although the blood level remained normal. The onset of treatment at this phase was more effective. On the other hand, a relatively higher level of blood ferritin made treatment less effective. Although the mechanism of such action is not well understood, it might be due to some interference by the higher concentration of blood ferritin.

In these experimental studies, $^{131}$I-labelled polyclonal antiferritin specifically localised to a s.c. tumour mass in concentrations sufficient to deliver a mean of approximately 4,125 cGy and 3,300 cGy to the tumours following infusion of 1,000 μCi of $^{131}$I-labelled polyclonal antibody in groups A and B respectively. These results are comparable to the radiation doses of 450 cGy following infusion of 500 μCi of $^{131}$I-labelled antiferritin antibody in a rat hepatoma model (Rostock *et al.*, 1983). We have several other ferritin-producing tumour lines of human hepatocellular carcinoma and neuroblastoma (unpublished data) which revealed unusually high levels of tumour ferritin within the first few weeks after inoculation although the serum level was normal.

An early process of development of a ferritin-producing tumour may not always be reflected by an increased level of ferritin in the peripheral blood. In our previous experimental study, we tried to target a very small inoculated tumour, ignoring the negative ferritin status of the blood. Administration of a very small dose of radioactive antiferritin (30 μCi per mouse) showed a clear image of the inoculated tumour following scintigraphy (Une *et al.*, 1989).

Nowadays, several imaging diagnostic methods are available for the detection of early primary or metastatic small lesions of human hepatocellular carcinoma but no single method has proved completely accurate (Oren *et al.*, 1986). Hepatic lesions less than 2.0 cm in diameter often give false negative results and are therefore difficult to detect (Lundstedt *et al.*, 1985). Therefore, immunodetection using radioactive antiferritin may provide a useful tool for the detection of small, or early, lesions of hepatocellular carcinoma especially in the high risk group of patients. Only 12–16% of the animals treated with 500 μCi of $^{131}$I-labelled antiferritin showed complete regression. Since the surviving tumour cells continue to express the ferritin antigen, improved results require an increase in antibody concentration in tumour relative to normal organs. In this experimental study, only a single dose of labelled antibody was given. The prolonged retention of antibody in tumour, compared to the concentration in blood and other organs, suggest that it should be possible to use multiple doses of antibody to achieve an increase in tumour concentration.

There have been reports that requirements for therapy with radiolabelled antibodies are different from those for imaging (Larson *et al.*, 1983; Leichner *et al.*, 1983; Rostock *et al.*, 1983). Our findings suggest a common requirement for treatment and for imaging, that is, increased concentration of tumour ferritin in the early phase of tumour growth.

Therefore, we conclude that it is possible to equate the antigen expression as a function of tumour growth time with localisation of antibody and thus with radiation dosimetry from $^{131}$I-labelled antiferritin antibody and therapeutic responses.

The author is a medical graduate from Mymensingh Medical College, under the University of Dhaka, Bangladesh, This work is submitted as his doctoral (Ph.D) thesis and was performed in Hokkaido University, Japan, while he studied in the graduate school of Medicine, being awarded a scholarship from the Ministry of Education, Japan.

The author gratefully acknowledges Prof J. Uchino for his support and suggestions; Dr Yuji Satoh for direct supervision and sincere cooperations; Dr T. Nojima for his guidance in pathological investigations and Drs K. Itoh and H. Arai for their assistance in the field of nuclear medicine.

**References**

BALDWIN, R.W. & PIMM, M.W. (1983). Antitumour monoclonal antibodies for radioimmunodetection of tumors in drug targeting. *Can. Med. Rev.*, **2**, 89.

CHRISTOPHER, C.B., KENNETH, A.K., ARTHUR, V.P., HOWARD, S. & IRWIN, D.B. (1985). Experimental radiotherapy of murine lymphoma with $^{131}$I-labeled anti-thy 1.1 monoclonal antibody. *Cancer Res.*, **45**, 1536.

COHEN, C., BERSON, S.D. & SHULMAN, G. (1985). Liver iron stores and hepatitis B antigen status. *Cancer*, **56**, 2201.

COHEN, C., BERSON, S.D., SHULMAN, G. & BUDGEON, L.R. (1984). Immunohistochemical ferritin in hepatocellular carcinoma. *Cancer*, **53**, 1931.

DILLMAN, L.T. (1969). Radionuclide decay schemes and nuclear parameters for use in radiation-dose estimation. *MIRD Pamphlet No. 4*, *The Society of Nuclear Medicine*.

EPENETOS, A.A., NIMMON, C.C. & ARKLIE, J. (1982). Detection of human cancer in an animal model using radiolabelled tumor associated monoclonal antibodies. *Br. J. Cancer*, **46**, 1.

KEW, M.C., TORRANCE, J.D., DERMAN, D. & 4 others (1978). Serum and tumor ferritins in primary liver cancer. *Gut*, **19**, 294.

KLEIN, J.L., NGUYEN, T.H., LAROQUE, P. & 7 others (1989). Yttrium-90 and iodine-131 radioimmunoglobulin therapy of an experimental human hepatoma. *Cancer Res.*, **49**, 6383.

KUWAHARA, T. (1980). Heterotransplantation of human hepatoma in nude mice – establishment of serially transplantable hepatoma. *Acta Hepatologica Japonica*, **21**, 303.

LARSON, S.M., CARRASQUILLO, J.A., KROHN, K.A. & 8 others (1983). Localization of $^{131}$I-labeled p97-specific Fab fragments in human melanoma as a basis for radiotherapy. *J. Clin. Invest.*, **72**, 2101.

LEICHNER, P.K., KLEIN, J.L., SIEGELMAN, S.S., ETTINGER, D.S. & ORDER, S.E. (1983). Dosimetry of $^{131}$I-labeled antiferritin in hepatoma: specific activities in the tumor and liver. *Cancer Treat. Rep.*, **67**, 647.

LIU, K.D., TANG, Z.Y., BAO, Y.M. & 4 others (1988). Radioimmunotherapy for hepatocellular carcinoma (HCC) using $^{131}$I-anti HCC isoferritin IgG: preliminary results of experimental and clinical studies. *Int. J. Radiat. Oncol. Biol. Phys.*, **16**, 319.

LUNDSTEDT, C., EKBERG, H., HALLDORSDOTTIR, A., TRANBERG, K.G. & STIGSSON, L. (1985). Angiography as a diagnostic, prognostic and therapeutic tool in liver metastases from a colorectal primary tumor. *Acta Radiol. Diagnos.*, **26**, 373.

MENARD, S., MIOTTI, S. & TAGLIABUE, E. (1983). Tumor radioimmunolocalization in the murine system using monoclonal antibodies. *Tumori*, **69**, 185.

MOROZ, C., LANTSBERG, Z., SELA, O. & 5 others (1989). Radioimmunodetection of tumors with monoclonal antiplacental ferritin antibody: preliminary results. *Oncology*, **46**, 35.

MOSHAKIS, V., MCILHANNEY, R.A.J. & RAGHABAN, D. (1981). Localization of human tumor xenografts after *i.v.* administration of radiolabeled monoclonal antibodies. *Br. J. Cancer*, **44**, 91.

NITTSU, Y., OHTSUKA, S. & KOHGO, Y. (1975). Hepatoma ferritin in the tissue and serum. *Tumor Res.*, **10**, 31.

ORDER, S.E., KLEIN, J.L. & LEICHNER, P.K. (1986). Radiolabeled antibody in the treatment of primary and metastatic liver malignancies. Recent results. *Cancer Res.*, **100**, 307.

OREN, J.W., FOLSE, R., KRAUDEL, K.L. & LEWIS, D.B. (1986). The preoperative liver scan and surgical decision-making in patients with colorectal cancer. *Am. J. Surg.*, **151**, 452.

PRESSMAN, D. (1957). Radiolabeled antibodies. *Ann. N.Y. Acad. Sci.*, **69**, 644.

ROSTOCK, R.A., KLEIN, J.L., LEICHNER, P.K., KOPHER, K.A. & ORDER, S.E. (1983). Selective tumor localization in experimental hepatoma by radiolabeled antiferritin antibody. *Int. J. Radiat. Oncol. Biol. Phys.*, **9**, 1345.

ROSTOCK, R.A., KOPHER, K.A. & BAUER, T.W. (1985). Factors that affect antiferritin localization in four rat hepatoma models. In: *Cancer drug delivery*, Vol. 2. Mary Ann Libert Inc.: New York, p. 139–45.

TANG, Z.Y., LUI, K.D., GUO, Y.D. & 7 others (1986). Tumor imaging and targeting therapy for hepatocellular carcinoma. Preliminary results and experimental studies. *Chin. Med. J. Engl.*, **99**, 855.

TANG, Z.Y., LIU, K.D., BAO, T.M. & 7 others (1990). Radioimmunotherapy in the multimodality treatment of hepatocellular carcinoma with reference to second look resection. *Cancer*, **65**, 211.

UNE, Y., SATOH, Y., ALAM, S., HATA, Y. & UCHINO, J. (1989). Study on the accumulation and antitumor effect of antiferritin antibody against human hepatoma implanted in nude mice. *Drug Delivery Systems*, **4**, 74.

ZHANG, X., KLEIN, J.L. & ORDER, S.E. (1988). Quantitative comparison of tumor dose of radiolabeled monoclonal antibodies in an experimental tumor model. *Antibody Immunoconjugates Radiopharmaceuticals*, **1**, 35.